# Harnessing digital health data for person‐centred HIV prevention monitoring: a survey of national health information systems

**DOI:** 10.1002/jia2.70039

**Published:** 2025-10-08

**Authors:** Shona Dalal, Bradley Mathers, Dominik Stelzle, Lilly M. Nyagah, Francis Agbo, Dennis Annang, Saiprasad Prabhakar Bhavsar, Stone Mbiriyawanda, Bongiwe Mhlanga, Tshepo Molapo, Lowrence Moro, Peter Mudiope, Linea Ngwali, Mwiche Siame Nyirenda, Isabel Sathane, Rajatashuvra Adhikary, Monica Alonso Gonzalez, Polin Chan, Annette Gerritsen, Kiyohiko Izumi, Giorgi Kuchukhidze, Antons Mozalevskis, Georges Perrin, Ahmed S. Alaama, Madidimalo Tebogo, Annette Verster, Daniel Low‐Beer

**Affiliations:** ^1^ Global HIV Hepatitis and STIs Programmes World Health Organization Geneva Switzerland; ^2^ Kirby Institute, University of New South Wales Sydney New South Wales Australia; ^3^ Ministry of Health, National AIDS, STI and Viral Hepatitis Control Program Nairobi Kenya; ^4^ National Agency for the Control of HIV/AIDS Abuja Nigeria; ^5^ Ghana AIDS Commission Accra Ghana; ^6^ Ministry of Health and Family Welfare New Delhi India; ^7^ Ministry of Health Lilongwe Malawi; ^8^ Ministry of Health Mbabane Eswatini; ^9^ Department of Health Johannesburg South Africa; ^10^ Ministry of Health Juba South Sudan; ^11^ Ministry of Health Kampala Uganda; ^12^ Ministry of Health Windhoek Namibia; ^13^ Ministry of Health Lusaka Zambia; ^14^ Ministry of Health Maputo Mozambique; ^15^ World Health Organization, India Country Office New Delhi India; ^16^ Pan‐American Health Organization Washington DC USA; ^17^ World Health Organization Regional Office for South‐East Asia New Delhi India; ^18^ The Global Fund Geneva Switzerland; ^19^ World Health Organization Regional Office for the Western Pacific Manila Philippines; ^20^ World Health Organization Regional Office for Europe Copenhagen Denmark; ^21^ World Health Organization Regional Office for Africa Brazzaville Republic of the Congo; ^22^ World Health Organization Regional Office for the Eastern Mediterranean Cairo Egypt; ^23^ World Health Organization Gaborone Botswana

**Keywords:** data, surveillance, testing, HIV, prevention, health information systems

## Abstract

**Introduction:**

Measuring HIV prevention impact is challenging because prevention is started and stopped as needed, and individual‐level data availability has been suboptimal or not collected. WHO's 2022 *Consolidated guidelines on person‐centred HIV strategic information* aim to bridge this gap by recommending a minimum dataset for HIV prevention monitoring.

**Methods:**

We surveyed the availability of 42 HIV prevention data elements collected on an individual from WHO's recommended minimum dataset in 21 countries’ national health information systems during a Prevention Outcome Monitoring Workshop held in September 2024 in Gaborone, Botswana. Over 150 participants representing ministries of health and programme implementers from 21 countries in Africa and Asia, as well as representatives from global organizations, attended. National HIV prevention managers completed the survey covering: registration (client demographics, use of unique identification, key population status), HIV testing, HIV prevention and vertical transmission. Data element availability determined which prevention indicators each country could calculate. Additionally, we describe global data on the use of unique identification for key populations.

**Results:**

Of the 21 attending countries, 18 completed the survey. Fifteen countries (83%) used unique identification in their national health information systems. All 18 countries collected HIV testing data elements, while 14–18 countries (78–100%) collected those for vertical transmission. However, prevention data availability varied widely. Different data elements on pre‐exposure prophylaxis (PrEP) and post‐exposure prophylaxis (PEP) were collected by 13–17 (72–94%) countries, condoms by 15 (83%) and voluntary medical male circumcision by 11 (61%) countries. Data elements on harm reduction were available in 4–6 countries among 8–10 countries providing services. While all countries could calculate HIV testing indicators, around 90% could for vertical transmission, 50–94% for PrEP/PEP and 40–75% for harm reduction. Only two countries could calculate linkage to prevention, which incorporates all prevention interventions. Kenya was the only country that collected all recommended person‐centred data elements. Overall, up to 37 of 105 reporting countries had a nationally harmonized personal unique identification method for key populations.

**Conclusions:**

Data building blocks for HIV prevention exist in most national health information systems. Aligning these systems with global standards offers potential to further strengthen person‐centred HIV prevention monitoring.

## 1 INTRODUCTION

Although HIV incidence continues to decline, it is far from the global target of < 370,000 new infections in 2025, with 1.3 million people acquiring HIV in 2024 [1]. Particularly affected are key populations (men who have sex with men, sex workers, people who inject drugs, trans and gender diverse people, and people in prisons and other closed settings) and their partners in all world regions, and adolescent girls and young women in sub‐Saharan Africa [2, 3]. Reaching individuals at elevated risk of HIV acquisition with their choice of effective HIV prevention is critical to reducing incidence [4]. Yet, monitoring HIV prevention programmes to identify gaps in coverage and opportunities for improvement has been a challenge due to the episodic nature of HIV prevention need and use, a lack of individual‐level data, and the common practice of aggregating information across interventions, which make composite indicators difficult to interpret.

The evolution of health information systems towards digital, person‐centred (or individual‐level) data can be harnessed for HIV prevention. Digitizing routine programmatic data and using it for HIV prevention can help identify individuals at elevated risk of HIV acquisition, generate prevention demand and determine intervention uptake. By comparison, aggregate counts of prevention services delivered do not allow for deduplication and make it difficult to assess whether programme coverage is over‐saturating a small population or has a broader reach across a priority population. Since routine data only capture those who access health services, they need to be adjusted for population size to derive population‐level estimates.

Routine national monitoring systems are essential for data collection for the HIV response. The World Health Organization's (WHO) 2022 *Consolidated guidelines on person‐centred HIV strategic information* aim to strengthen the analysis and use of routinely collected data at each stage of a person‐centred cascade of care for HIV and related infections [5]. For the first time, a minimum dataset for HIV prevention was recommended to measure interventions received and health outcomes among individuals seeking prevention. The minimum dataset describes specific data elements to be collected that indicate the date and the intervention received for each client. For example, the date an individual was prescribed pre‐exposure prophylaxis (PrEP), the product prescribed and the volume dispensed. Such standardization of data collection tools enables calculation of indicators and is a critical component in developing a comprehensive monitoring system. Additionally, unique identification facilitates monitoring individuals across various services, enabling the measurement of prevention outcomes over time.

In this paper, we present the findings from a survey on national HIV prevention monitoring conducted among 18 participating countries to assess the availability of prevention data elements in national health information systems. We reviewed the potential for calculating HIV prevention indicators based on these data to inform current and future programme planning.

## 2 METHODS

We shared a survey containing 42 data elements required for individual‐level, person‐centred HIV prevention monitoring as described in the WHO Strategic Information Guidelines [5], with 21 countries attending a HIV Prevention Outcome Monitoring Workshop in September 2024 in Gaborone, Botswana. There were over 150 participants with representatives of ministries of health and programme implementers from 21 countries in Africa and Asia, as well as representatives from global organizations. National HIV prevention managers completed the survey, administered as a series of yes/no questions on specific data elements. We categorized the data elements into four sections covering registration (client demographics, use of unique identification, key population status), HIV testing, HIV prevention and vertical transmission. The data elements were then used to determine which prevention indicators could be calculated.

Additionally, we present global data on the use of unique identification for key populations from the publicly available Global AIDS Monitoring National Commitments and Policies Instrument. This is an annual national survey conducted by UNAIDS, WHO and UNICEF [6]. We analysed 10 questions from the survey, two for each key population.

WHO guidelines suggest two options for indicator denominators: (1) Service‐level denominators, which include all people who have sought services. These denominators use available data and are actionable at the individual or facility level, but exclude people who have not sought services and may be at risk for HIV. (2) Population‐based denominators that require the use of population size estimation or modelling to determine the population at risk and, therefore, provide a comprehensive picture of prevention coverage. In this analysis, we used service‐level denominators for simplicity, as population‐based denominators were not always available.

No data was collected on individuals which required ethical clearance.

## 3 RESULTS

### 3.1 Review of WHO‐recommended minimum dataset for HIV prevention

Eighteen of 21 attending countries completed the review of data elements (Botswana, Cameroon, Cote d'Ivoire, Eswatini, Ghana, India, Indonesia, Kenya, Malawi, Mozambique, Namibia, Nigeria, Philippines, South Africa, South Sudan, Tanzania, Uganda, Zambia). The availability of data elements varied considerably, with the widest variation observed in HIV prevention data elements (Table 1). Among 18 reporting countries, 15 used at least one unique identifier – 10 countries used a national identification number, of which nine countries additionally used a national health/ programme/insurance identifier, and only Botswana used no other identifier. Five countries without national identifiers used at least one of the following: national health/programme/insurance identifiers. Most countries collected the date of birth (*n* = 16); one used estimated age. In categories for sex or gender, five countries collected options other than male/female. Under key populations, most countries collected categories for men who have sex with men and sex workers (*n* = 16; 89%); 12 countries (67%) did for trans and gender diverse people and people in prisons and other closed settings; 14 countries (78%) did so for people who inject drugs; and 10 countries (56%) collected all key populations. All 18 countries collected HIV testing data elements, while 14 (78%)–18 (100%) countries collected different data elements on vertical transmission.

**Table 1:** Availability of WHO‐recommended minimum data elements [5] for HIV prevention monitoring in national surveillance systems

		Countries reporting (*n* = 18)	
Area/intervention	Data element	Number	Percent
Unique identification method	Any form of unique identification^a^	15	83%
	National unique identifier	10	56%
	National health identifier	9	50%
	Programme identifier (such as ART number)	12	67%
	National health insurance identifier	9	50%
Demographics	Date of birth^a^/age	17	94%
	Sex^a^	16	89%
Key population member	Sex worker	16	89%
	Men who have sex with men	16	89%
	Trans and gender diverse people	12	67%
	People who inject drugs	14	78%
	People living in prisons and other closed settings	12	67%
HIV testing	HIV test sample date^a^	18	100%
	HIV test result^a^	18	100%
Prevention referral	Elevated risk for HIV acquisition (based on risk factors)	10	56%
PrEP	Date PrEP prescribed, including initial prescription^a^	17	94%
	Date PrEP dispensed^a^	16	89%
	PrEP product prescribed^a^	13	72%
	Volume of PrEP product prescribed or dispensed (e.g. number of pills, number of devices)^a^	14	78%
PEP	Date PEP prescribed^a^	15	83%
	Date PEP course completed (ascertained at follow‐up)^a^	14	78%
NSP	Date injecting equipment provided^a^	4	22%
	Number of needles–syringes provided^a^	5	28%
OAMT	Date OAMT initiated^a^	5	28%
	Date OAMT dose received^a^	6	33%
	Date OAMT take‐away dose(s) dispensed^a^	6	33%
	Date of first maintenance dose received^a^	6	33%
	Date of loss to follow‐up or OAMT stopped^a^	6	33%
VMMC	VMMC procedure conducted	11	61%
	VMMC procedure date^a^	10	56%
Condom programming	Condom provision date^a^	15	83%
	Condoms distributed (at service delivery points or national warehouse)	15	83%
Vertical transmission of HIV, hepatitis B and syphilis	ANC contact date^a^	14	78%
	Date of first ANC visit^a^	16	89%
	Maternal HIV status at first ANC visit^a^	17	94%
	Date of ART initiation^a^	16	89%
	Already on ART at first ANC visit^a^	17	94%
	Newly on ART during pregnancy^a^	18	100%
	HIV‐exposed infant or child^a^	17	94%
	HIV‐exposed infant or child test result of HIV assay 1^a^	17	94%
	Final diagnosis of HIV‐exposed infant^a^	16	89%
	Maternal syphilis test result	17	94%
	Hepatitis B diagnosis	15	83%

Abbreviations: ANC, antenatal care; ART, Antiretroviral therapy; NSP, needle and syringe programme; OAMT, opioid‐agonist maintenance therapy; PEP, post‐exposure prophylaxis; PrEP, pre‐exposure prophylaxis; VMMC, voluntary medical male circumcision.

^a^ Data element included in WHO‐recommended minimum dataset described in Consolidated guidelines on person‐centred HIV strategic information [5].

HIV prevention data elements collected consistently included PrEP, post‐exposure prophylaxis (PEP), condoms and voluntary medical male circumcision (VMMC) (Table 1). Up to one‐third of all countries collected data elements on harm reduction such as needle and syringe programmes (NSPs) and opioid‐agonist maintenance therapy (OAMT), including India, Kenya, Malawi, Mozambique, Tanzania and Uganda. When restricted to countries known to provide NSP (*n* = 10) or OAMT (*n* = 8) services, data element availability increased to 40–75% of countries.

### 3.2 Global analysis of use of unique identification for key population prevention services

A total of 105 countries responded to questions on unique identification for key population prevention services in the Global AIDS Monitoring National Commitments and Policies Instrument in 2023. Overall, 37 countries had a nationally harmonized personal unique identification method for men who have sex with men, 35 countries for sex workers, 26 countries for trans‐ and gender diverse people, 22 for people who inject drugs and 15 for other priority populations, such as people in prison or migrants (Table 2). The most common method for identifying and removing duplicate health information across key populations was a combination of routinely collected personal identifying information. This was followed by the use of HIV‐specific unique identifiers and then national unique identifiers. The use of biometric identification was uncommon.

**Table 2:** Methods of unique identification used by countries for key population prevention services

	Number of countries using different types of unique identifiers					
Key population	Nationally harmonized personal unique identifier	National unique identifier	Combination of personal identifying information	HIV‐specific unique identifier	Biometric	Other
Men who have sex with men	37	11	12	7	1	6
Sex workers	35	10	12	7	1	5
Trans and gender diverse people	26	10	7	5	0	4
People who inject drugs	22	7	5	4	1	5
Other priority population	15	5	4	3	1	2

### 3.3 Assessment of indicators

The prevention indicators that we assessed required between one and nine data elements for calculation: one data element for simple counts and nine for the more complex linkage to the prevention indicator, which incorporates referral to all prevention services. Some HIV testing indicators could be calculated by all (*n* = 18) countries, with the exception of the linkage to prevention indicator, which could only be calculated by two countries (Table 3). Indicators for vertical transmission were possible to assess for up to 94% (*n* = 17) of countries. Harm reduction indicators were the lowest, ranging from 40% (NSP coverage and regular NSP access, *n* = 4) to 75% (OAMT coverage, *n* = 6) of countries able to calculate those indicators. Kenya was the only country with all data elements needed to calculate all indicators, including suggested gender, age and key population disaggregations.

**Table 3:** HIV prevention‐related indicators, required data elements, and the number and proportion of countries that can analyse the indicators based on individual‐level data

Indicator number [5]	Indicator name	Required data elements	Countries that could analyse indicator (*n* = 18)	
			Number^a^	Proportion
**HIV prevention**				
PRV.1	Condoms distributed	Condoms distributed	15	83%
PRV.2	Total PrEP recipients	Date PrEP prescribed	17	94%
PRV.3	PrEP coverage	At elevated risk for HIV acquisition; HIV test date; HIV test result; date PrEP prescribed	9	50%
PRV.4	Volume of PrEP prescribed	Date PrEP prescribed; volume PrEP prescribed	14	78%
PRV.5	Number of PEP recipients	Date PEP prescribed	15	83%
PRV.6	PEP completion	Date individual completed PEP course (ascertained at follow up)	14	78%
PRV.7	HIV in PEP recipients	Date individual completed PEP course (ascertained at follow up); HIV test date; HIV test result	14	78%
PRV.8	NSP coverage	Date injecting equipment provided; key population member type	4^a^	40%
PRV.9	Regular NSP access	Date injecting equipment provided; key population member type	4^a^	40%
PRV.10	Needles‐syringes distributed	Number of needles‐syringes provided	5^a^	50%
PRV.11	OAMT coverage	Date OAMT prescribed; key population member type	6^a^	75%
PRV.12	Total person‐years on OAMT	Date OAMT initiated; date of loss to follow‐up or OAMT stopped	5^a^	63%
PRV.13	OAMT minimum duration	Date OAMT initiated; date of first date maintenance dose received	5^a^	63%
PRV.15	VMMC	VMMC procedure date	10^a^	56%
**HIV testing and knowledge of status**				
HTS.1	PLHIV know status	HIV status	18	100%
HTS.2	HTS volume and positivity	HIV test date; HIV test result	18	100%
HTS.3	Individuals testing positive for HIV	HIV test date; HIV test result	18	100%
HTS.7	HTS linkage to prevention	HIV test date; HIV test result; elevated risk for HIV acquisition; date PrEP prescribed; date PEP prescribed; date injecting equipment provided; date OAMT initiated; VMMC procedure date; condom provision date	2	11%
HTS.8	HIV retesting coverage	HIV test date; HIV test result; elevated risk for HIV acquisition	10	56%
**Vertical transmission**				
VER.2	Early infant diagnosis coverage	HIV test date; HIV‐exposed infant or child	16	89%
VER.6	Final outcome of PMTCT	Final diagnosis of HIV‐exposed infant or HIV‐exposed infant or child; HIV test date; HIV test result	16	89%
VER.7	HIV prevalence among women attending ANC	ANC contact date; HIV status; HIV test date; HIV test result	17	94%

*Note*: All indicators and metadata are described in detail in WHO's Consolidated guidelines for person‐centred HIV strategic information [5].

Abbreviations: ANC, antenatal care; HTS, HIV testing services; NSP, needle andsyringe programme; OAMT, opioid‐agonist maintenance therapy; PEP, post‐exposure prophylaxis; PLHIV, people living with HIV; PrEP, pre‐exposure prophylaxis; VMMC, voluntary medical male circumcision.

^a^ Analysis restricted to 10 countries with NSP programmes and eight countries with OAMT programmes.

## 4 DISCUSSION

Operationalizing person‐centred data for HIV prevention requires data building blocks in health information systems for calculating prevention indicators to monitor services and address gaps. Overall, our findings show that data element collection for HIV testing, condom usage, PrEP, PEP, VMMC and vertical transmission was generally good across countries, although some gaps remain. In contrast, harm reduction services were only offered in 8 (OAMT)–10 (NSP) countries, 4–6 of which collected the relevant data elements. Harm reduction data collection is influenced by existing laws and policies that criminalize drug use, therefore, limiting service provision and reducing data availability. Our results indicate that with a few strategic additions, prevention data in national systems can be significantly enhanced. Political will and robust healthcare infrastructure are crucial for building and maintaining effective monitoring systems for HIV prevention. International development assistance cuts as seen in 2025 can undermine these efforts, leading to gaps in service delivery, reduced data security and weaker programmes. Ongoing investment and leadership are essential for building robust systems and preventing programmatic reversals.

A critical aspect of prevention monitoring is effectively addressing elevated risk for HIV. Our review suggests that the collection of clinical and behavioural information on factors associated with HIV acquisition is possible within health information systems, with all countries collecting at least six prevention data elements. However, the gaps seen highlight a need for additions. Health information systems must also adapt to include new interventions such as lenacapavir and long‐acting formulations in electronic medical records, and enable disaggregation by service type. Given that an individual's need for HIV prevention changes over time, frequent updates to information collected during routine visits and other points of contact are necessary to maintain the relevance of prevention interventions offered [5, 7]. Offering HIV prevention services both to those identified with risk factors for HIV, as well as those who self‐identify and request prevention, will ensure that individuals receive the prevention services they need.

Key populations face a disproportionate burden of HIV, sexually transmitted infections (STIs) and viral hepatitis (particularly hepatitis B and hepatitis C), and good quality data are critical for understanding and improving services for them [2]. Our findings show that while several countries collect data on key populations, a comprehensive approach including all key populations is lacking. Only 10 countries collected data on all key populations. The National Commitments and Policies Instrument results further underscore the importance of unique identification methods for key population prevention services. The most common method for identifying and removing duplicate health information was a combination of routinely collected personal identifying information, which is not recommended by WHO for secure unique identification [5]. Rather, a unique identifier for health should not include any personally identifying information. The criminalization and stigmatization of behaviours associated with key populations contribute to barriers in accessing health services and complicate data collection efforts [2]. There can be serious negative consequences if data indicating an individual has engaged in an activity that is stigmatized or criminalized is not kept confidential. People may be less inclined to reveal this information or to engage in services if they fear information about them might be compromised.

The most frequently used unique identifier reported by countries in our analysis was a programme identifier for HIV; 10 used a foundational national identifier. While programme‐specific identifiers are useful for monitoring individuals in HIV care and treatment, they may not provide the cross‐disease linkage necessary to identify HIV prevention need – for example individuals who have had an STI in the previous year [8, 9, 10]. Furthermore, the use of a national identifier rather than a unique identifier for health raises the potential for exposure of sensitive health information in settings unrelated to healthcare where that identifier is used, and may inhibit disclosure of HIV risk factors to healthcare workers. Digital health data systems must foster public trust through transparent governance and be protected by legal frameworks to ensure the confidentiality and security of client information [5, 11, 12, 13, 14]. Maintaining the separation of sensitive behavioural data from personally identifying information, even when linking to other clinical datasets, is recommended to protect individual privacy [5].

While data are crucial for granular and timely analyses, the quality and reliability of these data can vary significantly across different settings. Data quality depends on how diligently information is captured by health service providers and health information systems, and how it is cleaned, reviewed and used. As countries continue using individual‐level routine facility data, their quality will inevitably improve. Implementing appropriate unique identifiers will help eliminate duplicate patient records and improve data accuracy. Innovations such as artificial intelligence can further aid in data quality checks. However, de‐identification protocols, restricted access and transparency in algorithmic processes are needed. Engaging and training data stewards, and regularly evaluating data quality, will be important to ensure that collected information remains accurate, reliable and actionable.

The results from our review are from a few countries, predominantly in sub‐Saharan Africa, and, therefore, are not representative of all countries globally. We also did not assess data or their quality in the national health information systems, but rather focused on the building blocks of a robust data system for HIV prevention. However, our results show the types of prevention data commonly collected in high HIV burden countries and identify data collection gaps.

## 5 CONCLUSIONS

Strengthening person‐centred health information systems is crucial for improving person‐centred care and effective programme planning. By addressing the gaps in prevention data availability and ensuring the confidentiality and security of sensitive information, HIV programmes can enhance the offer of prevention interventions and support better programme monitoring and health outcomes.

## COMPETING INTERESTS

No conflicts of interest for any authors have been declared.

## AUTHOR CONTRIBUTIONS

SD conceptualized the manuscript and wrote the first draft. BM and LMN contributed to writing and reviewing. DS analysed data and reviewed and edited the manuscript. FA, DA, SPB, SM, BM, TM, LM, PM, LN, LMN, MSN, and IS contributed data for analysis and reviewed and edited the manuscript. RA, MAG, PC, AG, KI, GK, AM, GP, ASA, MT, AV, and DLB reviewed and edited the manuscript.

## FUNDING

No funding was received for this manuscript.

## DISCLAIMER

The authors alone are responsible for the views expressed in this article, and they do not necessarily represent the views, decisions or policies of the institutions with which they are affiliated.

## Data Availability

Global AIDS Monitoring National Commitments and Policies Instrument data is available publicly from https://aidsinfo.unaids.org/. The de‐identified data that support the findings of this study are available from the corresponding author upon reasonable request.
